# *In situ* X-ray nanotomography of metal surfaces during electropolishing

**DOI:** 10.1038/srep15257

**Published:** 2015-10-15

**Authors:** Maryana I. Nave, Jason P. Allen, Yu-chen Karen Chen-Wiegart, Jun Wang, Surya R. Kalidindi, Konstantin G. Kornev

**Affiliations:** 1 Department of Materials Science and Engineering, Clemson University, Clemson, SC 29634, USA; 2 School of Materials Science and Engineering, Georgia Institute of Technology, Atlanta, GA 30332, USA; 3 Photon Sciences Directorate, Brookhaven National Laboratory, Upton, NY 11973, USA; 4 Department of Mechanical Engineering, Georgia Institute of Technology, Atlanta, GA 30332, USA

## Abstract

A low voltage electropolishing of metal wires is attractive for nanotechnology because it provides centimeter long and micrometer thick probes with the tip radius of tens of nanometers. Using X-ray nanotomography we studied morphological transformations of the surface of tungsten wires in a specially designed electrochemical cell where the wire is vertically submersed into the KOH electrolyte. It is shown that stability and uniformity of the probe span is supported by a porous shell growing at the surface of tungsten oxide and shielding the wire surface from flowing electrolyte. It is discovered that the kinetics of shell growth at the triple line, where meniscus meets the wire, is very different from that of the bulk of electrolyte. Many metals follow similar electrochemical transformations hence the discovered morphological transformations of metal surfaces are expected to play significant role in many natural and technological applications.

## Introduction

Physico-chemical phenomena occurring at the solid–liquid interface play key role in many areas of science and technology. Electrochemical polishing of metals belongs to this class of problems and it poses many challenges associated with the complexity of the electrochemical reactions at the solid-electrolyte interface. In this method, a metal specimen of interest is connected to the anode and is submersed into the electrolyte. A cathode was placed in the same electrolyte and when an external voltage differential is applied, electrochemical reactions cause the anode specimen to corrode^1^. Electrochemical polishing finds different industrial applications, including electrochemical machining and electropolishing of metals and alloys in medical industry (shaping and finishing medical instruments and implants), in food industry (processing blades and foils), in microelectronics (making circuits and microtrenches), etc^1^. Among different applications dealing with electrochemical polishing of metals, nanotechnology applications are, probably, the most demanding, attractive, and challenging^2^. Electropolishing is used in fabrication of sharp tips for scanning tunneling microscopy^3^, atomic force microscopy^4^, field ion microscopy^5^, Raman spectroscopy^6^, nanolithography^7^,^8^, micro/nano welding^9^,^10^, microcircuitry and sensorics^11^,^12^ and many biomedical applications^13^,^14^,^15^. This method is able to provide the wire tips of different metals^16^,^17^,^18^,^19^,^20^,^21^,^22^ with the radius of curvature of the apex varying from a few nanometers to submicrons.

A detailed understanding of kinetics of electrochemical reactions, the role of electrolyte oversaturation, precipitation of complex compounds and growth of oxide and hydrate films and porous layers is critical for the technology development and control of the metal microstructure. The solid/vapor interface has been actively researched with the advanced imaging and characterization techniques^23^,^24^. The solid/liquid and solid/liquid/vapor interfaces call for the development of new techniques requiring a special care for distinguishing different effects occurring simultaneously at the interfaces especially at the nanoscale level^25^,^26^.

In this work, we undertake a tour de force around a technique for the nanoscale *in situ* visualization of electrochemical processes accompanying electropolishing of tungsten wires. Tungsten was chosen due it’s high Young modulus and melting point making it an attractive candidate for a variety of applications in biomedicine^13^,^14^, scanning probe microscopy^4^,^6^,^12^,^16^, nanolithography^8^ and others. To satisfy the requirements of *in situ* X-ray imaging and to accommodate a tungsten wire, electrolyte, and circuitry, we designed and built a special electrochemical cell. The wire was submersed vertically into an aqueous solution of potassium hydroxide. In order to suppress the evolution of hydrogen, electropolishing is typically conducted at a high anodic potential (~10 V for tungsten)^27^,^28^,^29^,^30^ . This regime leads to the wire necking, a kind of field induced morphological instability which is difficult to control^30^. We used the regime of low voltage electropolishing^14^ applying only 2 ± 0.5 V. The low voltage electropolishing offers rich opportunities for making sharp probes with different tip morphologies^14^.

When the wire is partially submersed into electrolyte, the metal/electrolyte interface cannot be visualized using optical microscopes: meniscus rises up the wire precluding the observations. The X-ray imaging allows one to penetrate through the optically dense media and air/liquid interfaces. Thus, the morphological features of growing solid films can be studied in real time. Using these novel techniques, we have investigated the kinetics of formation of different surface films on the wire surface. Applying a rigorous image processing, we discovered that the tungstate oxide films growing next to the metal phase can be shielded from electrolyte by another layer of complex compounds. This shielding effect promotes the uniform and stable polishing of the entire submersed portion of the wire leading to the formation of the centimeter long needle^14^. This is the main difference between the low voltage electropolishing and conventional high voltage electropolishing generating only hundred micron long taper^30^. The rates of film formation and dissolution are proven to be different in the bulk of electrolyte and in the vicinity of triple line where three air/electrolyte/metal surfaces meet. This discovery calls for reconsideration of the fundamentals of electropolishing, and sets new challenges for the explanation of the observed corrosion phenomena at the triple line.

## Methods

### Materials

A 85% potassium hydroxide (KOH) was purchased from Alfa Aesar^®^ Company. Potassium hydroxide aqueous solution with concentration of 1 M was prepared by dissolving KOH flakes in deionized water. The solution was stirred and left to settle for one hour before the experiment. A stainless steel wire with 500 μm diameter and 10 mm length was used as cathode. Tungsten wires with diameters of 75 μm and 125 μm, with 99.95% purity, were purchased from Advent Research Materials Ltd and used as anodes.

### Transmission X-ray microscopy

We employed the transmission X-ray microscopy (TXM) on the tungsten wires at room temperature. The beamline X8C of National Synchrotron Light Source, Brookhaven National Laboratory was employed^31^.

**Figure 1:**
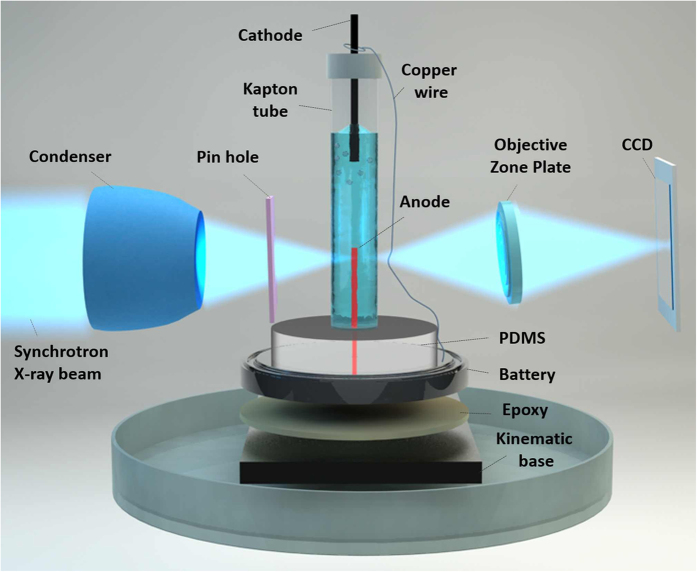
Schematic of the sample positioning on the transmission X-ray microscope (TXM). A tungsten wire was immersed in a Kapton tube filled with the electrolyte. The wire was connected to a battery and was placed on the sample stage. Rotating the stage, one acquires the necessary images. Created by Ella Marushchenko.

The schematic of the TXM microscope is shown in Fig. 1, where the X-ray beam with the 8960 eV energy is focused by the capillary condenser on the sample (i.e., tungsten wire). A Fresnel zone plate is used as an objective lens to form an image onto the CCD detector (2048 × 2048 pixels) with the camera binning 2 × 2 pixels or 4 × 4 pixels with a single pixel size of 20 nm. The images were continuously collected with the 1 s exposure time. The field of view was 40 × 40 μm^2^. After the measurements, the background was normalized and an automatic alignment using the run-out correction system was applied.

This technique offers a resolution at a sub-30 nm level in 2D and sub-50 nm in 3D^31^. It also allows continuous collection of images from the stationary sample to form *in situ* 2D movies. For tomography, the images can be collected at multiple angles, while the sample is rotated on a special stage around its axis. 1011 number of TXM projections were collected for one tomographic measurement. A standard filtered back-projection reconstruction algorithm was used to construct the 3D images^32^.

### Electrochemical cell

Figure 1 shows a schematic of the electrochemical cell used in experiments under the X-ray beam. A kapton capillary tube with the inside diameter of 2.05 mm and the length of 20 mm was imbedded from both sides into the thermally cured Polydimethylsiloxane (PDMS) disks. A tungsten wire was threaded through the needle gage 34, from the bottom side through the PDMS disk into the kapton capillary. Using epoxy, the other side of the tungsten wire was attached to the positive side of the battery. This method allowed one to have about 3 mm long piece of the tungsten wire with the diameter of 125 μm inside the tube. The X-ray beam was focused at the 40 μm by 40 μm spot situated 300 μm bellow the free end of the wire. A stainless steel wire with diameter of 500 μm was pierced through the PDMS disk from the top side of the capillary tube and was attached with an insulated copper wire to the negative side of the battery. Due to the size lamination of the kinematic base in Fig. 1, we used the NUON^®^, coin silver oxide batteries generating the 1.55 V voltage. This voltage was sufficient to perform the low voltage electropolishing and observe the growth of the tungsten oxide and hydrate films on the tungsten surface. Moreover, employing these batteries, we made the setup self-contained and mobile.

To start and finish the electrochemical reaction on demand, the copper wire was mechanically connected/disconnected with the cathode using a manipulator arm. In this setup, the cathode and anode were interchangeable. Next, the battery with the tube was glued with epoxy to the kinematic base and placed into the Petri dish to prevent any spills on the TXM stage. Using the 1 ml syringe with a gage 27 needle, the electrolyte was injected into the tube from the top through the pierced hole in the PDMS disk.

In one series of experiments, Fig. 1, the tungsten wire was completely submersed into the electrolyte and the meniscus separating the electrolyte from the air was located above the free end of the wire. This setup was designed to study the morphological changes of the tungsten/electrolyte interface in the bulk of electrolyte.

In order to analyze the morphological changes of the wire surface near meniscus, when the wire was partially submersed in the electrolyte, we modified the setup by adding one more kapton tube. The tubes were connected by a channel to form a single U-tube. The anode was placed into one leg of the tube and the cathode was placed into the other leg. This design enabled stabilization of the meniscus and eliminated the influence of the hydrogen generated at the cathode. These two sets of experiments provided new insights onto the effects associated with the free surface of meniscus^14^,^30^ and separate them from those occurring in the bulk of electrolyte.

### Image processing

#### First method

**Figure 2:**
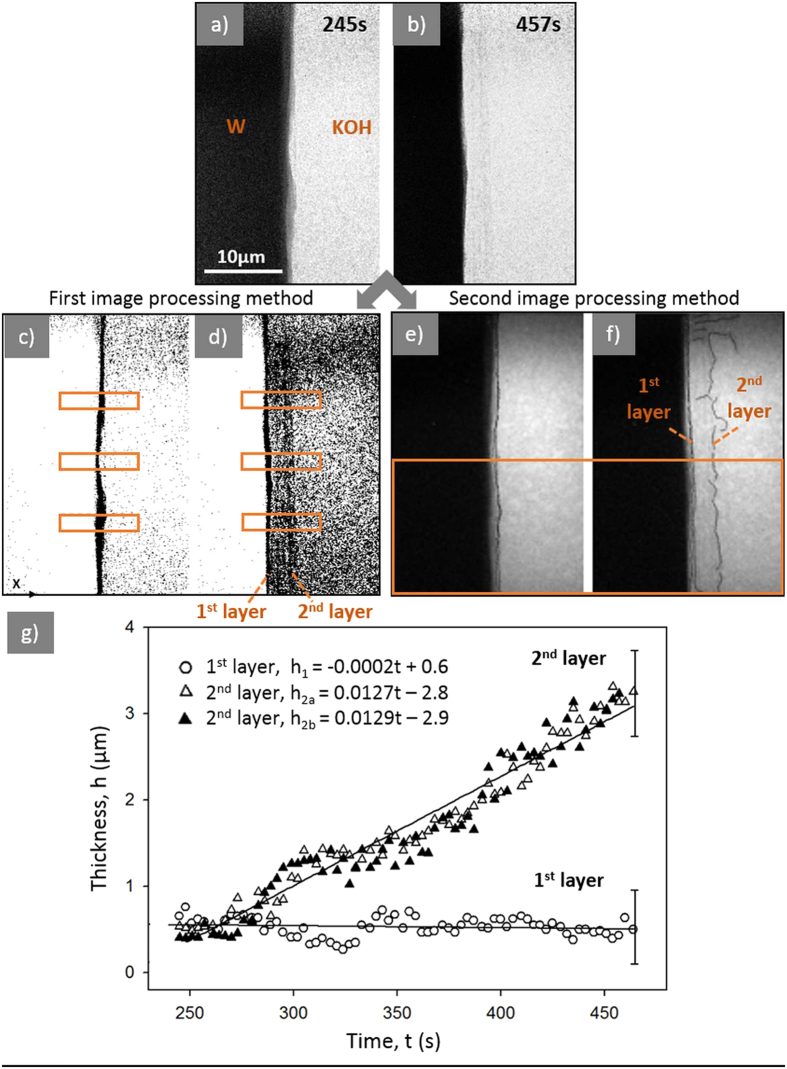
The morphological changes of the wire surface during electropolishing at the voltage difference E = 1.55 V: (a), (b) a sequence of the original *in situ* images showing the layered structure of the tungsten surface and the change of the layer thickness as the reaction progresses; (c), (d) the same sequence of images processed with ImageJ (NIH) to track the change of the layer thicknesses with time using the First method; (e), (f) the sequence of images processed with MATLAB^®^ using the Second method (numbers 1 and 2 correspond to Interface 1 and Interface 2, and the boxed regions identifies the segment of data used for average thickness calculations); (g) the layers thicknesses as a function of time with the error bars corresponding to the highest standard deviation in the experiments. Open circles and triangles were extracted using the First method of image processing and closed triangles were calculated using the Second method of imaging processing. Both results show a good correspondence with each other.

The original RGB images (Fig. 2(a,b)) were first converted into the 8-bit images, and then into the binary black and white images using thresholding (Fig. 2 (c,d)). To determine the rate of change of the layer thicknesses, three 10 μm × 2 μm boxes shown in Fig. 2 (c) were analyzed using Plot Profile function. The thicknesses, h, of the layers as a function of time, t, were extracted and plotted in Fig. 2 (g). The error bars were estimated based on the variation in the thickness layer between the three measured dashed boxes.

#### Second method

As an extension of the above simple image analyses, we have also conducted a more in-depth image analysis that is based on gradients of the gray scale level in the images. While the thresholding approaches are better suited for identifying the dominant constituents in the image, the gradient-based image analyses are expected to be more accurate in identifying the interfaces between the constituents. In these protocols, the 8-bit images were first smoothed using Gaussian filters and the gradients in the horizontal direction (normal to the anode surface) were computed using the fourth-order central finite difference scheme Image differentiation was applied both spatially (i.e., differentiation horizontally across an image) and temporally (i.e. differentiation across a particular pixel from one image in time to the next) to improve the contrast in the images. The backgrounds of the spatially differentiated images were removed by subtracting the image complement leaving the gradient maxima that correspond to the interfaces between layers (i.e. the regions of maximum rate change). Taking the complement of the remaining images and using thresholding followed by an image erosion operation, the interfaces were identified for each image^14^. MATLAB’s built-in Canny edge detection algorithm was applied to temporally differentiated images to clearly identify the interfaces between the different layers.

The interfaces computed from each method were then integrated into a single image. All images were then converted to a binary (black and white) image and the number of pixels between different interfaces, identified as Interface 1 and Interface 2 (Fig. 2 (e,f)), including the pixels that represent the interfaces, were computed. The raw data images and images with identified interface overlays corresponding to measurements well below the meniscus are found in Figure S3 while those corresponding to measurements around the meniscus are found Figure S4.

## Results

### The wire is completely immersed into the electrolyte

A sequence of the X-ray images in Fig. 2 (a,b) demonstrates the morphological changes of the tungsten surface during electropolishing. The tungsten wire appears black. As the electrochemical reaction progresses, the black region becomes separated from the electrolyte (shown gray) by two distinguishable layers (Fig. 2 (d)). The first layer that is sitting on top of the tungsten appears darker, hence it is denser. The second layer which is directly exposed to the electrolyte is much brighter, hence it is less dense. The analyzed image borders are fixed in space as shown in the boxes of Fig. 2 (c) therefore, one can follow the electropolishing kinetics by tracing the position of the dark boundary and the boundaries of associated layers. From the sequence in Figure S2, one infers that even though both layers are moving continuously, the thickness of the darker layer does not change significantly as compared to that of the brighter layer. Once formed, the first layer thickness remains almost unchanged while the second layer thickness increases with time.

The observations presented above lead to the following inferences: (i) the reaction rates are high at the two interfaces (tungsten-first layer and the first layer-second layer) seen in the images as the gradients in the grayscale (related to density) are highest at these interfaces, (ii) the reaction rates within the two layers are somewhat uniform within the layer (as no significant gradients were observed within each layer), and (iii) the reaction rates are very low in the second layer (no significant change in the grayscale in the second layer with time). In other words, two different kinds of reactions must be occurring at the two interfaces, separated by a more or less a uniform distance between them. Possible explanations for these two types of reactions will be discussed later.

**Figure 3:**
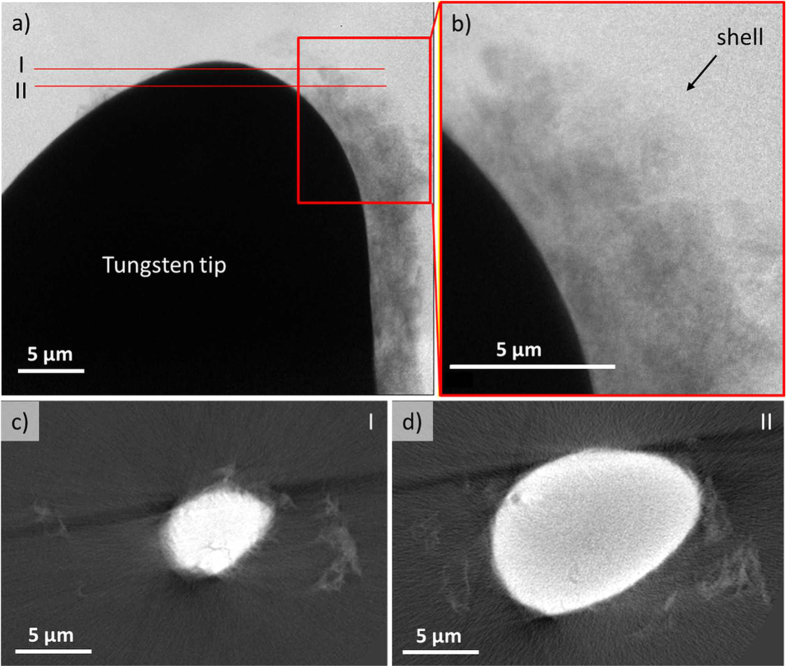
TXM tomography: (**a**) 2D image of the tungsten tip with the shell, (**b**) enlarged section from the image (**a**) of the shell; (**c**), (**d**) cross-sections from 3D reconstruction of images collected during tomography: I and II red lines depicted in the image (**a**) indicate the cross-section location.

*Ex situ* tomography was conducted on the 125 μm tungsten needle electropolished under the 2 V applied potential in the 2 M KOH aqueous solution. After the electropolishing procedure was finished, the sample was carefully withdrawn from the solution in order to keep the shell intact. However, because the shell was weakly adhered to the wire, the majority of the shell was washed out by the liquid during the wire withdrawal. The sample was dried in the air and stored in the Petri dish before the tomography was performed. Figure 3 shows the results of the tomography scan. The tungsten tip was only partly covered with a shell. The shell appears to have an irregular porous structure (Fig. 3 (c,d)). As evident from a Supplementary movie, this shell is highly permeable and allows adjacent liquid film to flow through it^14^.

Thus, the results of X-ray imaging suggest that the electropolishing at low voltages progresses by formation of two distinguishable layers, the first layer adjacent to tungsten is denser and seems solid, while the second layer appears porous. Figure 2 (g) summarizes the results of analysis of the growth kinetics of these two layers (shown with open circles and triangles); the layer thicknesses were analyzed from three boxes shown in Fig. 2 (c). From the plot it can be seen that the thickness of the first layer remains the same through the reaction, but the thickness of the second layer increases linearly with time.

As mentioned earlier, it is often advantageous to analyze the images using gradient-based analyses of the gray scale level as they can provide more reliable information on the spatial locations of the interfaces (Fig. 2 (e,f)). The sequence of images in Figure S3 (a) show the tungsten wire evolving with time while corresponding images in Figure S3 (b) show two interface overlaid on the original images. We will call the first Interface 1 and the second Interface 2. The reason for these names will be given below. The number of pixels between Interface 1 and Interface 2, including the pixels on the interface lines (Figure S3 (c)), were computed and are shown in Fig. 2(g) as the average layer thickness (shown with solid triangles) as a function of time. This plot shows good correspondence with the layer thicknesses as a function of time calculated using the first method of image processing.

### The wire is partially submersed into the electrolyte

**Figure 4:**
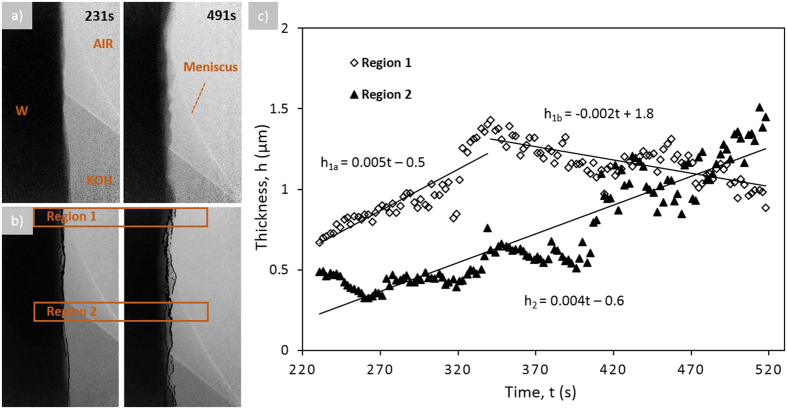
The morphological changes of the wire surface partially immersed in the electrolyte during electropolishing at the voltage difference E = 1.55 V: (**a**) a sequence of the *in situ* images showing the layered structure of the tungsten surface and the change of the layer thickness as the reaction progresses, (**b**) the same sequence of images with overlaid Interface 1 and Interface 2 (the boxed regions identify the segment of data used for average thickness calculations), (**c**) The average thickness of the layer for the tungsten wire partially submersed in electrolyte as a function of time calculated using imaging analysis methods for the boxed region identified in Figure S4 (**c**) (diamonds represent the average layer thickness for Region 1 while the triangles represent the average layer thickness for Region 2).

**Figure 5:**
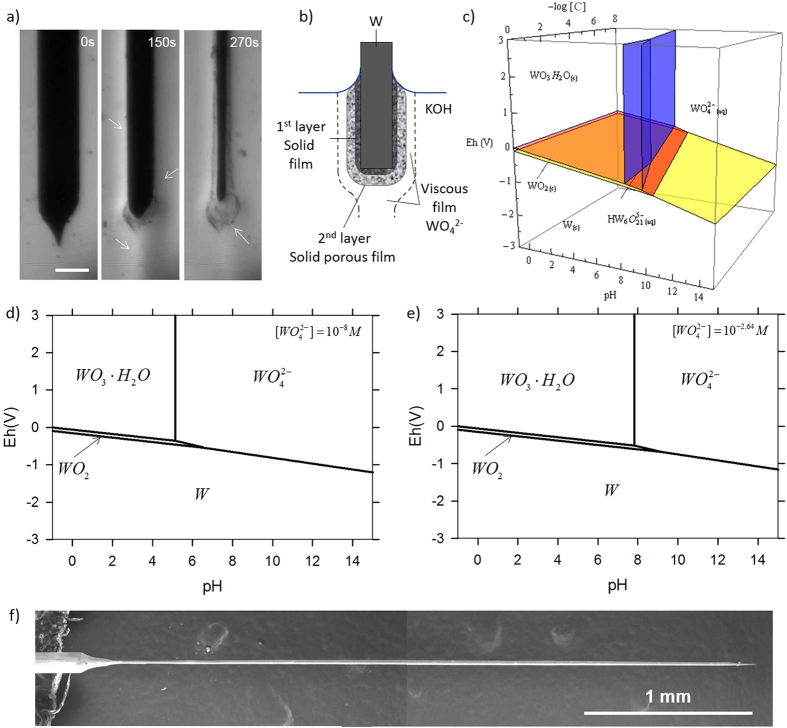
(**a**) Formation of a porous layer where the arrows on the 150 s frame point the viscous film and the arrow on the 270 s frame marks a porous film^14^ and the probe (f) obtained at 2 V; (b) the schematic of the layer hierarchy: electrolyte—highly viscous immiscible film—porous layer – dense layer of tungsten compound – tungsten wire; (c) the 3D Pourbaix diagram for tungsten in aqueous solution; (d), (e) the 2D cross-sections of the 3D Pourbaix diagram taken at the concentration of soluble species. 
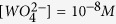
, 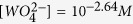 respectively.

When the wire is partially submersed into the electrolyte and as electropolishing proceeds, meniscus slides down the wire and dewets the wire surface. Electropolishing at low voltages causes formation of a porous shell as discussed in ref. 14 and is shown in the Supplementary movie and Fig. 5 (a,b). Several layers under the air/liquid interface grow and evolve with time. These layers fold, crawl, and wrinkle forming bumps and grooves. In our analysis, we follow only the changes of thickness of the entire shell not looking at its detailed internal morphology. Two regions at the wire surface were tracked: regions 1 and 2 in Fig. 4 (b). Region 1 exists only within about 340 seconds and then gets undressed by the sliding meniscus exposing this part of the wire surface to the air. Region 2 corresponds to the immersed part of the wire which stays inside the electrolyte for the entire time of electropolishing.

The second method of image processing was then repeated for the section of tungsten wire partially submersed in the electrolyte. As before, the number of pixels between Interface 1 and Interface 2, including the pixels on the interface lines (Figure S4 (c)), were calculated for the areas identified within the boxed regions. It should be clarified that Interface 2 exists above the meniscus line in Region 1 and below the meniscus line in Region 2. The computed average layer thickness as a function of time for Regions 1 and 2 is given in Fig. 4 (c).

This rigorous image processing allows us to state that the thickness of the entire film in region 1 first increases and after 340 seconds, when the meniscus slid down the wire exposing the wire surface to the air, the film thickness decreases. Contrary to that, the film thickness in region 2 keeps increasing during the experiment.

## Discussion

In order to get insight from the obtained data and to bring thermodynamic features to the electropolishing scenario, we constructed a 3D Pourbaix diagram, shown in Fig. 5 (c) (the details will be published elsewhere). It follows that tungstate ions and tungsten trioxide hydrate are the only stable species that can coexist with the aqueous electrolyte at the positive potentials *Eh*. The relation between voltage *Eh* and the voltage difference ΔE that is set between two electrodes, has been systematically studied in ref. 14. The results were perfectly fit by the formula 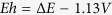. Therefore, we used this relation in our analysis of the Pourbaix diagram.

The tungstate ions are soluble in water and may be present in solution at the pH greater than pH 8. Bellow pH 8, the Pourbaix diagram does not allow these ions to occur and the tungsten ions cannot be free either. They would immediately form trioxide hydrate coexisting in equilibrium with the electrolyte.

According to these theoretical results, we can propose a scenario in which the pH value decreases from the bulk toward the tungsten surface. In the bulk of electrolyte where the pH value is large, e.g. pH 14 in our experiments, tungsten would be present only as a constituent of the tungstate ions. When we move closer to the wire surface, the pH value should decrease. Indeed, closer to the wire surface the negative tungstate ions get attracted to the wire. Therefore, the concentration of hydroxyl groups and protons should also decrease since the tungstate ions displace them. The closer the tungstate ions to the wire, the greater the chance for the electrolyte solution to be oversaturated with them. As shown in ref. 30, the solution undergoes a phase transition where a viscous film leaning to the wire surface has the properties distinct from that in the bulk of the electrolyte. Most important is the immiscibility of this viscous film. We therefore proposed that the viscous film was composed of an oversaturated solution of tungstate ions.

If the pH value decreases below some critical value, the solution of tungstate ions cannot exist anymore: these ions react with the hydroxyl groups to form tungsten trioxide hydrate. According to the Pourbaix diagram, tungsten trioxide hydrate can coexist with the electrolyte only as a solid phase (Fig. 5 c). Therefore, if one assumes that tungsten trioxide hydrate nucleates first as a submicron amorphous particle^33^, it would be attracted to the wire surface by the van der Waals forces. A similar assumption has been used for explanation of the pore formation in alumina membranes where boehmite 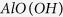 nanocrystals were hypothesized to play a significant role in building up the barrier layer^34^,^35^,^36^. In the favorable conditions, tungsten trioxide hydrate nuclei would build up a layer. Therefore, we hypothesize that the porous film observed at the wire surface is made of tungsten trioxide hydrate particles sintered together to form a porous solid film. This scenario satisfies the conditions of thermodynamic equilibrium of different species on the Pourbaix diagram. However, the proposed scenario does not follow a scenario of anodic electropolishing of metals^35^,^37^,^38^,^39^ which does not take into account thermodynamic constrains. These constrains are set on by the Pourbaix diagrams. To the best of our knowledge, while the two-layer model has been sugested for almost half a century ago, the current literature dealing with electropolishing does not address the thermodynamic limitations associated with incorporation of electrolyte anions such as 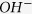 groups into the oxide structure^35^,^37^,^38^,^39^.

The Pourbaix diagram constructed for the aqueous electrolytes is unable to predict the chemical composition of the first layer separating tungsten from the tungsten trioxide hydrate layer. In the X-ray images, the first layer appears much denser than the second and we hypothesize that it is likely to be made of a solid tungsten oxide. This hypothesis is supported by multiple observations on tungsten surface^14^,^40^,^41^,^42^. However, we cannot predict what type of oxide can be stable in these conditions.

The proposed scenario provides a sufficient flexibility for observation of different regimes of electropolishing. The local concentration of tungstate ions and the pH values are interconnected through the Pourbaix diagram hence cannot be arbitrary changed. As we mentioned, the oversaturated solution of the tungstate ions cannot exist below some critical pH value. This value depends on the concentration of tungstate ions in solution. Figure 5 (d,e) demonstrate that at the beginning of the electrochemical polishing, when concentration of the tungstate ions is very small, for example, 
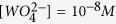
, the hydrate can be stable in the acidic environment, at the pH level lower than pH 5.14 (Fig. 5 (d)). However, once the concentration of the tungstate ions increases, for instance, up to 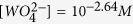, the hydrate becomes stable in more basic environment with the pH value lower than pH 7.82 (Fig. 5 (e)).

Therefore, at low voltages, electrochemical oxidation of tungsten in a potassium hydroxide solution proceeds with the formation of the following reaction products: tungsten oxide forming a layer on the tungsten surface, then tungsten trioxide hydrate layer which is sitting in contact with the tungsten oxide layer and separate it from the electrolyte. The layer of tungsten trioxide hydrate is porous and may contain aqueous solution of tungstate and potassium ions. In the bulk of electrolyte, we expect to observe only tungstate and potassium ions.

As follows from the EDS analysis in ref. 14 for the porous shell, the stoichiometric ratio of oxygen to tungsten at the surface of tungsten is about 2.4 suggesting that the 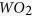 oxide is a best candidate to be the constituent of the oxide layer formed in this regime. The Pourbaix diagrams support this hypothesis. Indeed, for the positive potentials *Eh*, if we move from the bulk, where only tungstate ions can be found, toward the porous shell formed by the insoluble 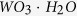 compound, we see that the only oxide which is able to coexist with this compound is the 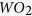 oxide. However, one has to assume that the applied potential has to drop at the oxide surface to make this phase coexistence possible. This reduction of electric potential at the metal surfaces has been documented in the literature^43^,^44^,^45^; it is most likely related to the complexity of the zone structure of tungsten dioxide which sits between metals and semiconductors.

This layering explains the X-ray images in Fig. 2 (a,b), where the dark part is tungsten; next to it is a thin gray layer representing the tungsten oxide and then a thick light gray layer represents the tungsten trioxide hydrate. Optical imaging was used to complement this scenario. Figure 5 (a) shows the kinetics of the layers formation. Before application of voltage, there was no flow of electrolyte around the tungsten wire. Once the voltage is applied, the tungsten surface appears surrounded by a denser fluid that flows downward (Fig. 5 (a) where the arrows at the 150 s image point a layer of a denser fluid). This electropolishing regime with a flowing film was analyzed in details in ref. 30, where the film was assumed to contain an oversaturated solution of tungstate ions. The Pourbaix diagram supports this hypothesis that the viscous film immiscible with the surrounding electrolyte represents a highly concentrated solution of tungstate ions that are formed as a result of the tungsten oxidation process. The 270 s image in Fig. 5 (a) illustrates the formation of a solid porous layer completely wrapping the tungsten wire. The thickness of this layer increases in time until the shell drops under its own weight (see the Supplementary movie). The analysis of movies suggests that a thin viscous film is always flowing down over the surface of this porous layer. Therefore, the layer of highly concentrated solution of tungstate ions fills the pores and spreads beyond the boundary of the porous tungsten trioxide hydrate film.

The existing models of formation of oxide layer and incorporation of polyelectrolyte anions into it with creation of the second layer assume that the thickness of oxide film increases linearly with time at a constant current^38^. Our experimental conditions provided almost constant current, but we did not observe any dependence of the thickness of oxide layer on time. This suggests that the amount of metal ions consumed in the oxidation reaction is exactly equal to the amount of oxide molecules removed from the oxide surface either in the form of dissociated ions or as new chemical complexes.

Results of the X-ray imaging and vigorous image analysis revealed a distinguishable kinetics of the shell growth at the triple line, where the electrolyte surface meets the wire surface. On the wire surface which was fully submerged into the electrolyte, the growth rate of the film was greater relative to the growth rate of the film sitting in the vicinity of the triple line. For example, the kinetic equations for the film growing on the fully submersed part of the wire in Fig. 2 (c) is given as 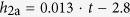 providing the rate 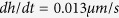. The kinetic equations for the different points on the film growing in the meniscus region of Fig. 4 (c) show about 3 times slow rate: for diamonds 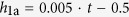 and for triangles 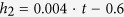.

Moreover, a decrease of the shell thickness in the vicinity of air/electrolyte/metal triple line as the wire surface is exposed to the air (in Fig. 4 (c), the trend is shown with diamonds, *h*_1b_) is surprising and requires a new thermodynamic and kinetics analyses and of diffusion/flow/evaporation/deformation in the porous layer occurring simultaneously at the triple line.

## Conclusions

Using X-ray imaging, nanotomography, optical microscopy, rigorous image processing and analysis of the Pourbaix diagrams, we studied evolution of micro and nanostructure of the surface of tungsten wire during electropolishing at low voltages. It is shown that the tungsten is shielded from the electrolyte by about 600 nm thick layer of tungsten oxide which forms right after the voltage application. This thickness does not change during ectropolishing. As electropolishing progresses, a complex tungsten containing compound, most likely tungsten trioxide hydrate, is formed on the oxide surface. This layer is porous and low permeable for the surrounding electrolyte. The thickness of this layer increases in time until the shell falls under its own weight. It is discovered that the thickness of porous shell decreases after meniscus slid down the wire.

## Additional Information

**How to cite this article**: Nave, M. I. *et al.* *In situ* X-ray nanotomography of metal surfaces during electropolishing. *Sci. Rep.* **5**, 15257; doi: 10.1038/srep15257 (2015).

## Supplementary Material

Supplementary Information

Supplementary Video for Figure 2

Supplementary Video for Figure 4

Supplementary Video for Figure 5a
