# Supplementary material for: In situ X-ray nanotomography of metal surfaces during electropolishing
Source: Sci Rep. 2015 Oct 15;5:15257. doi: 10.1038/srep15257 (PMC4606789; doi:10.1038/srep15257)
Supplement: Supplementary Information [file srep15257-s1.docx]

## *In situ* X-ray nanotomography of metal surfaces during electropolishing

Maryana I. Nave^1^, Jason P.Allen^2^, Yu-chen Karen Chen-Wiegart^3^, Jun Wang^3^, Surya R. Kalidindi^4^, and Konstantin G. Kornev^1^,*

^1^Department of Materials Science and Engineering, Clemson University, Clemson, SC 29634, USA

^2^School of Materials Science and Engineering, Georgia Institute of Technology, Atlanta, GA 30332, USA

^3^Photon Sciences Directorate, Brookhaven National Laboratory, Upton, NY 11973, USA

^4^Department of Mechanical Engineering, Georgia Institute of Technology, Atlanta, GA 30332, USA

*Correspondence and requests for materials should be addressed to K.G.K. (email: kkornev@clemson.edu)

Supporting Information


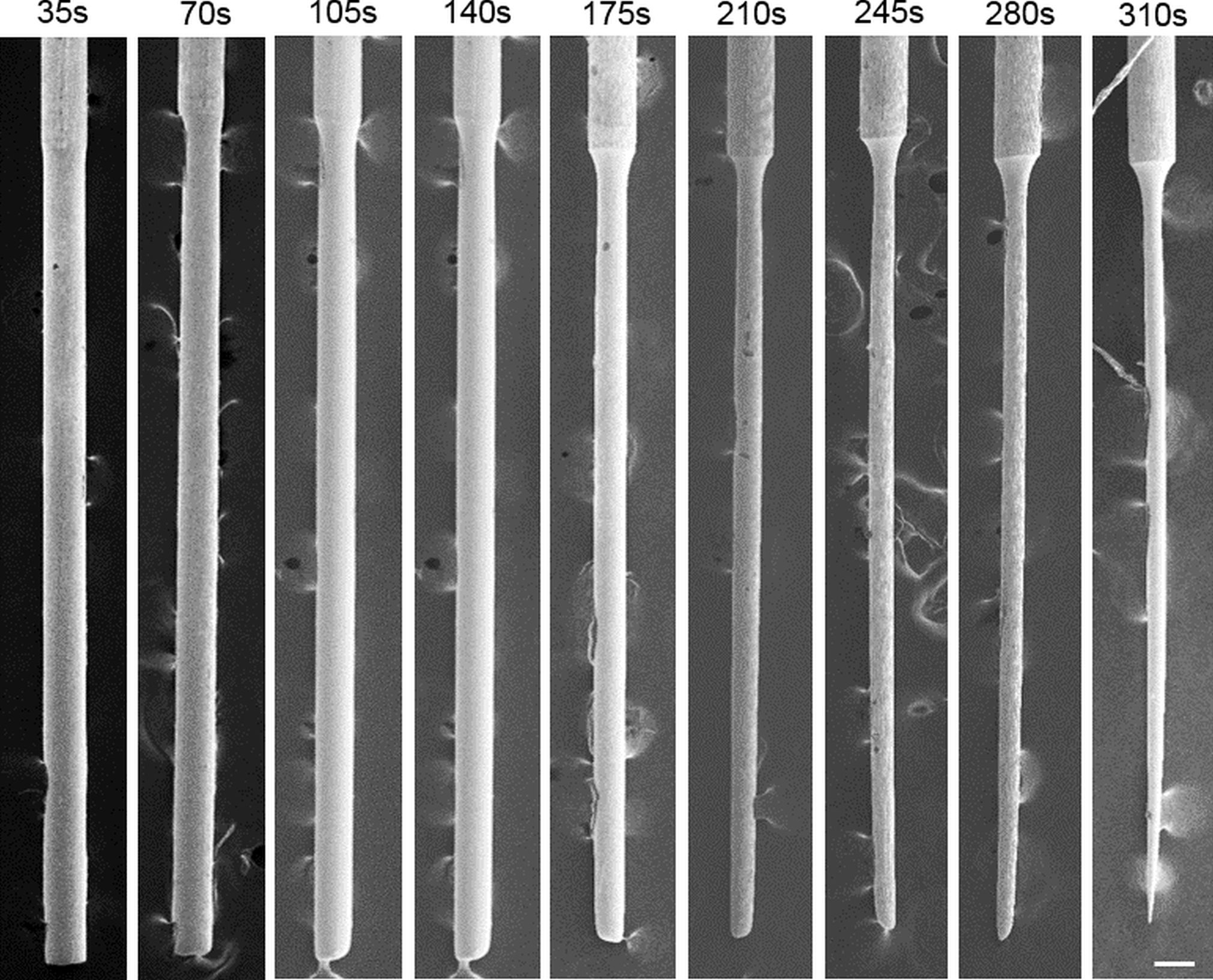


Figure S 1. Sequence of images showing change in diameter of the needle with time during low voltage electropolishing with 2V applied potential in 2M KOH electrolyte. Tungsten wires were removed from the electrolyte at different times and examined under SEM. The scale bar is 100 µm.


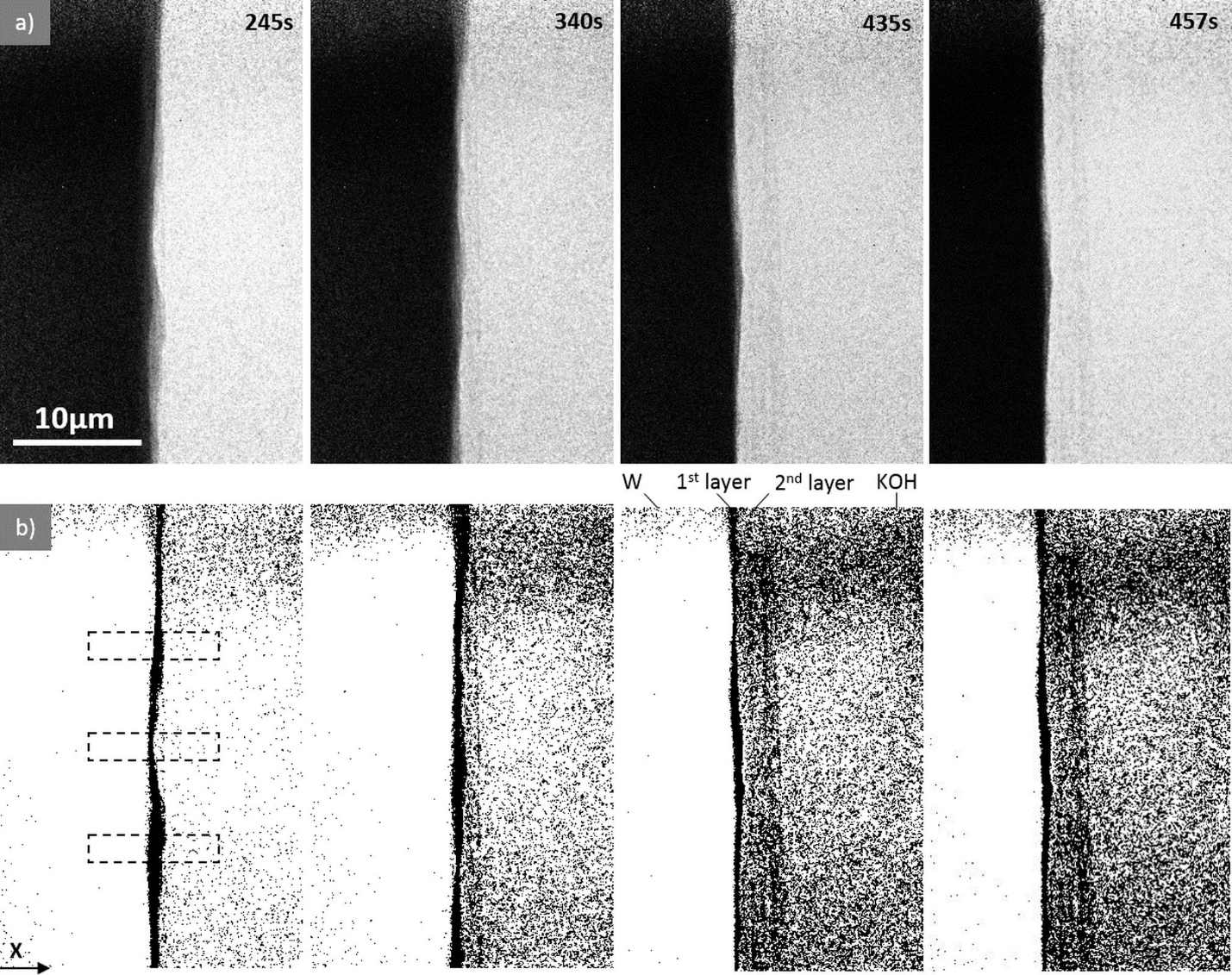


Figure S 2. The morphological changes of the wire surface during electropolishing at the voltage difference E=1.55V: (a) a sequence of the *in situ* images showing the layered structure of the tungsten surface and the change of the layer thickness as the reaction progresses, (b) the same sequence of images processed with ImageJ (NIH) to track the change of the layer thicknesses with time. The layer thicknesses from three dashed boxes were chosen for quantitate analysis.


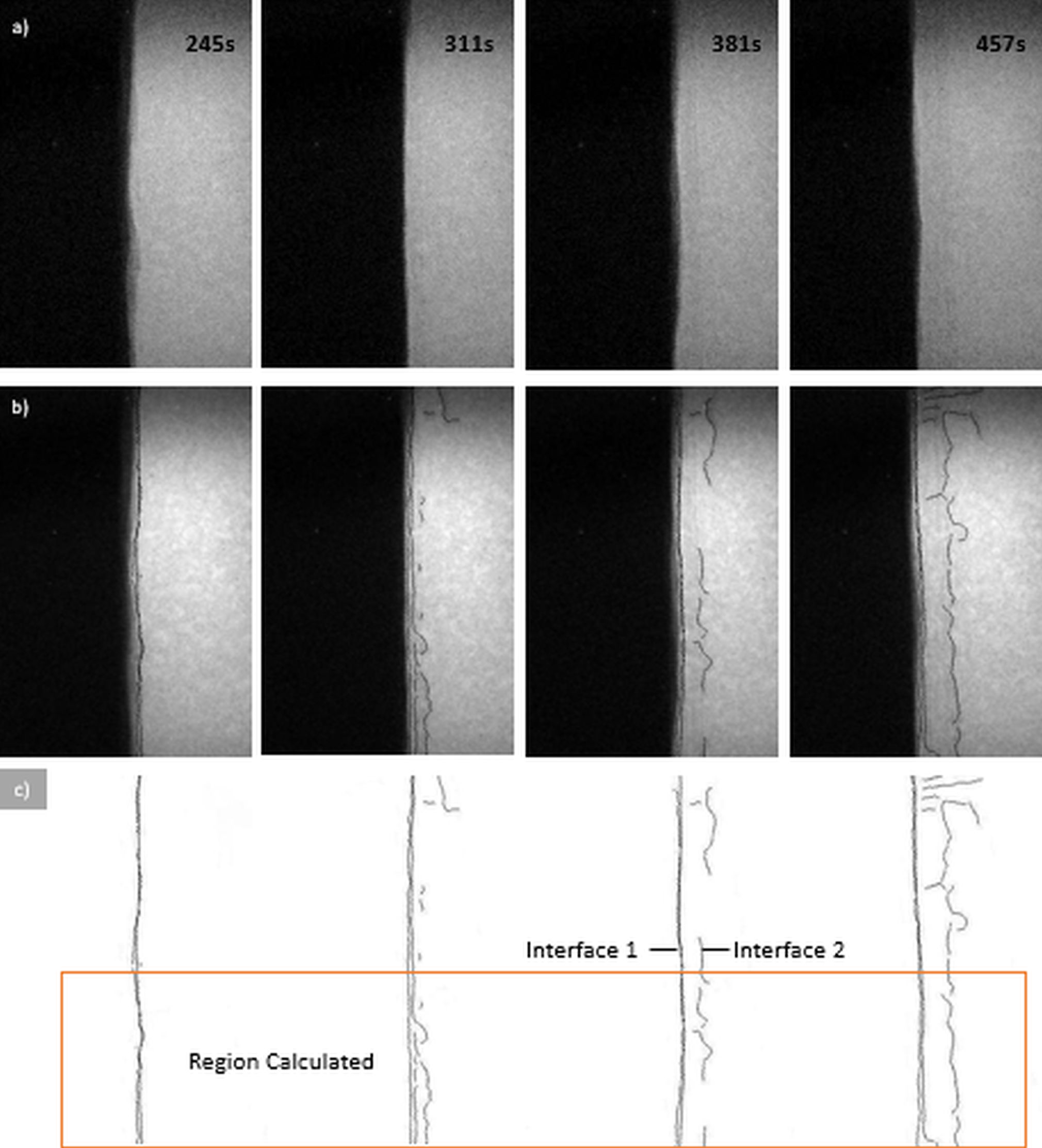


Figure S 3. The morphological changes of the wire surface completely immersed in the electrolyte during electropolishing at the voltage difference E=1.55V: (a) a sequence of the *in situ* images showing the layered structure of the tungsten surface and the change of the layer thickness as the reaction progresses, (b) the same sequence of images with overlaid Interface 1 and Interface 2, (c) the binary (black and white) representation of these interfaces denoted as Interface 1 and Interface 2. The boxed regions identifies the segment of data used for average thickness calculations.


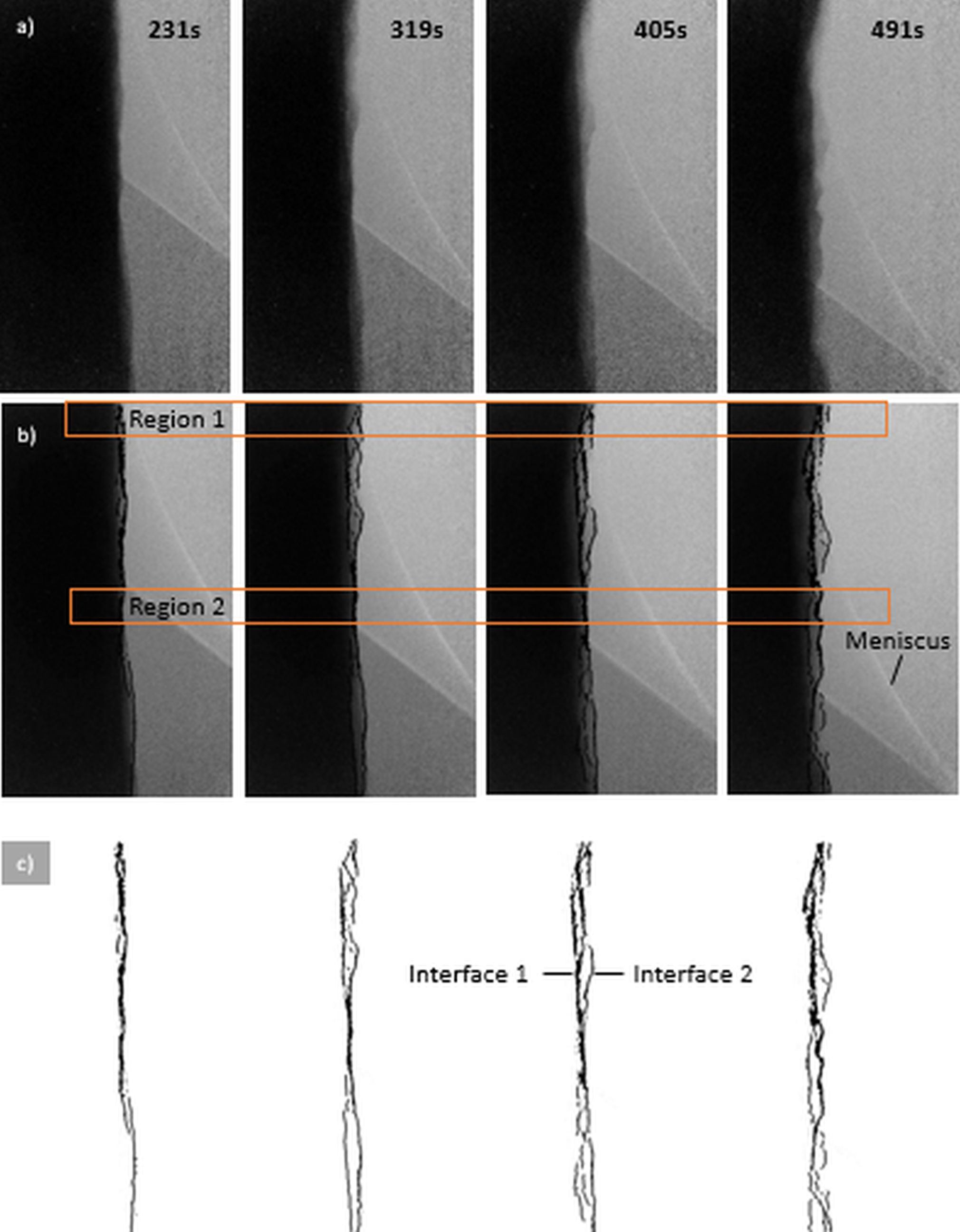


Figure S 4. The morphological changes of the wire surface completely immersed in the electrolyte during electropolishing at the voltage difference E=1.55V: (a) a sequence of the *in situ* images showing the layered structure of the tungsten surface and the change of the layer thickness as the reaction progresses, (b) the same sequence of images with overlaid Interface 1 and Interface 2, (c) the binary (black and white) representation of the Interface 1 and Interface 2. The boxed regions identify the segment of data used for average thickness calculations.

Using images from Figure S2 – Figure S4 the thicknesses of the layers as a function of time were extracted and plotted in Figure 2 (g) and Figure 4 (c).

In Figure 2 (g) the thickness plot for two layers versus time was constructed using the First and Second methods of the image processing techniques. These plots were fitted with the linear functions of time.

The first layer appears to have bumps and valleys at the initial moments of time. These morphological features followed the original defects and surface roughness of the virgin tungsten wire. Hence the growth of the first layer was very unstable due to continuous changes of the layer thickness observed at different spots along the wire surface. After about 360 seconds electropolishing, the thickness of the first layer has stabilized resulting in a smoother layer covering the entire wire surface. At the end of electropolishing, the thickness of the first layer appeared about 6 times smaller than that of the second layer. The best fit for the thickness of the first layer vs time was found as $h_{1}=-0.0002\cdot t+0.6$. The coefficient of determination for this approximation is $R^{2}=0.02$, i.e. it is close to zero suggesting a weak correlation of the film thickness with time. Therefore, the thickness can be considered time independent.

The thickness of the second layer vs time as extracted with the First method is described by the formula $h_{2a}=0.0127\cdot t-2.8$ with $R^{2}=0.96$. The Second method gives $h_{2b}=0.0129\cdot t-2.9$ with $R^{2}=0.94$. The growth rate of the second layer is independent of the image processing method and it exhibits a good predictability.

Region 1 in Figure 4 (c) yielded two trend lines: the first trend line describes the evolution of the second layer submersed into the electrolyte under the meniscus which was moving during electropolishing. The thickness changes as $h_{1a}=0.005\cdot t-0.5$ with$R^{2}=0.76$. The second trend line describes the same layer but after the meniscus has slid down and exposed this region to the air. The thickness changes as $h_{1b}=-0.002\cdot t+1.8$ with$R^{2}=0.57$.

Region 2 in Figure 4 (c) corresponds to the wire surface completely submersed into the electrolyte. This region yielded only one trend line with the continuously increasing thickness. The layer thickness changes with time as $h_{2}=0.004\cdot t-0.6$ with$R^{2}=0.84$.
